# Alarm pheromone and kairomone detection via bitter taste receptors in the mouse Grueneberg ganglion

**DOI:** 10.1186/s12915-017-0479-y

**Published:** 2018-01-18

**Authors:** Fabian Moine, Julien Brechbühl, Monique Nenniger Tosato, Manon Beaumann, Marie-Christine Broillet

**Affiliations:** Department of Pharmacology and Toxicology, Faculty of Biology and Medicine, University of Lausanne, Lausanne, CH-1011 Switzerland

**Keywords:** Olfaction, Grueneberg ganglion, Alarm pheromone, Predator scents, TAS2Rs, Danger detection

## Abstract

**Background:**

The mouse Grueneberg ganglion (GG) is an olfactory subsystem specialized in the detection of volatile heterocyclic compounds signalling danger. The signalling pathways transducing the danger signals are only beginning to be characterized.

**Results:**

Screening chemical libraries for compounds structurally resembling the already-identified GG ligands, we found a new category of chemicals previously identified as bitter tastants that initiated fear-related behaviours in mice depending on their volatility and evoked neuronal responses in mouse GG neurons. Screening for the expression of signalling receptors of these compounds in the mouse GG yielded transcripts of the taste receptors *Tas2r115*, *Tas2r131*, *Tas2r143* and their associated G protein α-gustducin (*Gnat3*). We were further able to confirm their expression at the protein level. Challenging these three G protein-coupled receptors in a heterologous system with the known GG ligands, we identified TAS2R143 as a chemical danger receptor transducing both alarm pheromone and predator-derived kairomone signals.

**Conclusions:**

These results demonstrate that similar molecular elements might be used by the GG and by the taste system to detect chemical danger signals present in the environment.

**Electronic supplementary material:**

The online version of this article (doi:10.1186/s12915-017-0479-y) contains supplementary material, which is available to authorized users.

## Background

**Fig. 1:**
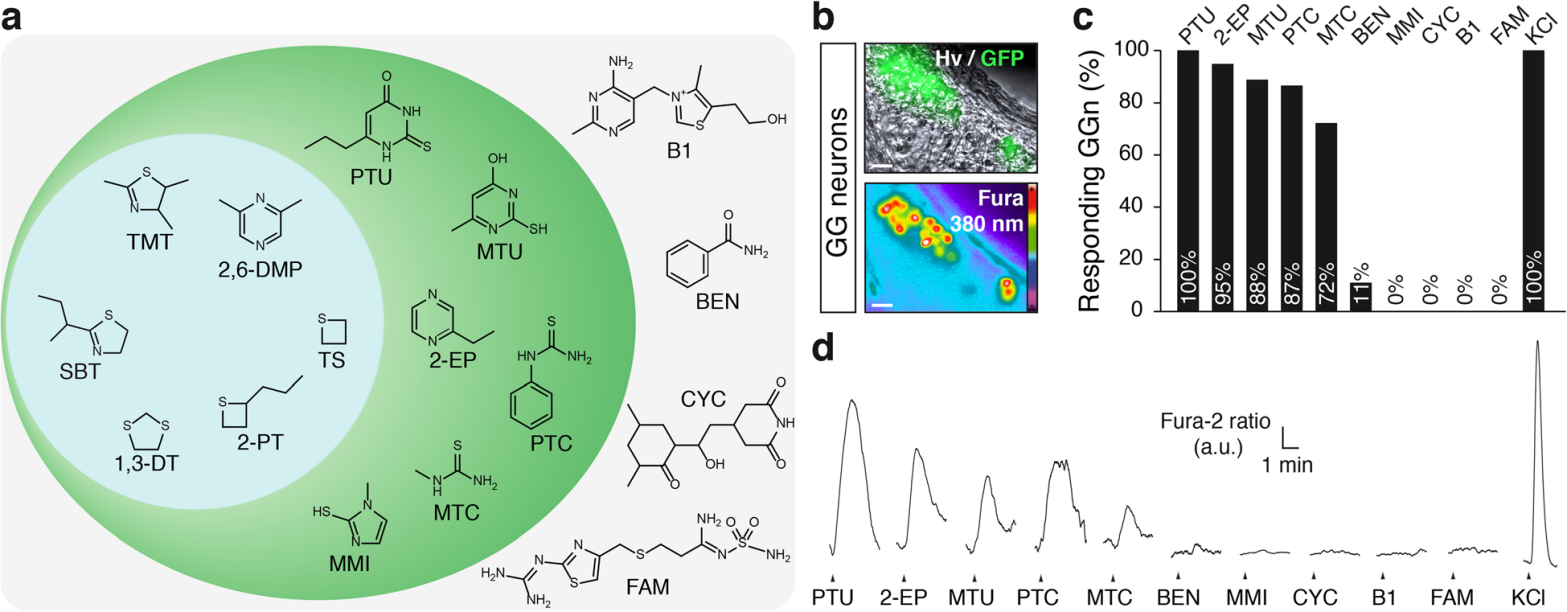
Mouse Grueneberg ganglion neurons are activated by bitter tastants structurally related to the olfactory danger signals. **a** The Grueneberg ganglion (*GG*) ligands (*blue panel*) 2-*sec*-butylthiazoline (*SBT*), 2,4,5-trimethylthiazoline (*TMT*), 2,6-dimethylpyrazine (*2,6-DMP*), 2-propylthietane (*2-PT*), trimethylene sulphide (*TS*) and 1,3-dithiolane (*1,3-DT*) are structurally related to a category of bitter ligands (*green panel*) composed of, for example, 6-propyl-2-thiouracil (*PTU*), 6-methyl-2-thiouracil (*MTU*), 2-ethylpyrazine (*2-EP*), phenylthiocarbamide (*PTC*), methylthiocarbamide (*MTC*) and methimazole (*MMI*). In the *external area* are bitter ligands with a chemical structure unrelated to GG ligands: benzamide (*BEN*), cycloheximide (*CYC*), vitamin B1 (*B1*) and famotidine (*FAM*). **b** GG coronal slice under Hoffman phase contrast (*hv*) from an olfactory marker protein-green fluorescent protein (OMP-GFP) mouse where GG neurons can be visualized due to their intrinsic GFP fluorescence (*upper panel*). Uptake of Fura-2 AM into GG neurons measured at 380 nm in a colour-coded map (*lower panel*). **c** Percentage of responding GG neurons (*GGn*) following perfusion of bitter ligands. PTU (34/34), 2-EP (37/39), MTU (16/18), PTC (13/15), MTC (13/18), BEN (2/18), MMI (0/18), CYC (0/9), B1 (0/18), FAM (0/20). **d** Representative calcium imaging recordings from GG neurons under perfusion of bitter ligands (1 mM). Control KCl (10 mM) perfusion is systematically performed at the end of experiment. In **b**, scale bars are 20 μm. In **c**, *n*/*x* = number of cells responding/number of cells tested and viable; obtained in a minimum of three different animals and six tissue slices for each tested cue. In **d**, fluorescence intensity Fura-2 ratio = F340/F380 is indicated by arbitrary units (*a.u.*); time is indicated by a horizontal bar

Survival of mammals in the environment depends on the ability of their sensory systems to process a variety of chemical molecules signalling danger [1]. The intraspecific alarm pheromones (APs) are, for example, molecules released by stressed or injured animals that induce in conspecifics stereotypic fear behaviours, such as an increased freezing time and a reduced walking distance [2]. Recently, we have discovered the structure of one mouse AP, 2-*sec*-butylthiazoline (SBT) [3] (Fig. 1a). This molecule is emitted in different stressful situations by mice of both sexes, and it induces the mentioned fear-related behaviours and also increases corticosterone levels in conspecifics. SBT is detected by the neurons of the Grueneberg ganglion (GG), an olfactory subsystem located at the tip of the nose [4–9]. SBT is a sulphated and nitrogenated heterocyclic compound which is closely related to predator scents that act as kairomones [10]. These interspecies communicating cues inform the preys of an impending danger. Indeed, kairomones are volatile molecules present in and emitted by the anal glands, urine or feces of mice predators, such as 2,4,5-trimethylthiazoline (TMT) from the fox [11], 2,6-dimethylpyrazine (2,6-DMP) from the bobcat [12], 2-propylthietane (2-PT) from the stoat [13] and trimethylene sulphide (TS) or 1,3-dithiolane (1,3-DT) from several Mustelidae [14]. We demonstrated earlier that these molecules also activate mouse GG neurons [3] and that they have, as well, the ability to induce fear-related behaviours in mice [3, 15–19]. These observations confer to the GG a double role, as it detects both intra- and interspecific danger signals, which bear a conserved chemical structure. The signalling pathways associated with the detection of these volatile olfactory danger signals are just beginning to be explored. The mouse GG expresses G protein-coupled receptors, including olfactory receptors [20–23], and a recent study has shown that a guanylyl cyclase-G receptor (GC-G) is an AP receptor in mice [24].

In our approach to identify the signalling pathways activated by danger signals, we took advantage of the chemical structure of previously identified GG ligands [3, 19] to screen chemical libraries, and we found a category of compounds already known as bitter tastants. We therefore set out to find if the ability to taste bitter chemicals, which is used by organisms to warn them against ingestion of toxic or spoiled food [25, 26], might also be adopted by the GG olfactory subsystem to detect structurally related chemicals. We found that exposing mice to these compounds initiated fear-related behaviours, and imaging studies showed their ability to evoke responses in mouse GG neurons. We further investigated the expression of potential receptors for these compounds in GG neurons and found three taste receptors (TAS2R115, TAS2R131, TAS2R143) and their associated G protein α-gustducin (GNAT3). Finally, heterologous expression studies support a role for TAS2R143 in the transduction of both APs and predator-derived kairomones. Thus, this bitter taste receptor present in mouse GG neurons might participate in the detection of these chemical danger cues.

## Results

### Identification of a new category of ligands for Grueneberg ganglion neurons

Using as bait the chemical structures of the mouse AP SBT and of the predator-derived kairomones [3, 19], which are sulphated and nitrogenated heterocyclic compounds, we identified a new category of putative GG ligands that possess a chemically related structure (Fig. 1a). We found among them analogues of low-weight molecules composed of a single heterocyclic ring, such as the uracil derivates 6-propyl-2-thiouracil (PTU) and 6-methyl-2-thiouracil (MTU), the pyrazine derivate 2-ethylpyrazine (2-EP), molecules bearing a urea or carbamide group such as phenylthiocarbamide (PTC) and methylthiocarbamide (MTC) and, finally, the imidazole derivate methimazole (MMI). This category of molecules has been identified previously as bitter tastants, as these molecules are able to activate several members of heterologously expressed human or mouse bitter taste receptors (TAS2Rs) [27–30].

We then tested these ligands on acute tissue slices of GG from olfactory marker protein-green fluorescent protein (OMP-GFP) mice, where GG neurons could be visualized due to their GFP expression [4, 31, 32] and loaded with the ratiometric calcium dye Fura-2 AM [4] for sets of calcium imaging experiments to detect evoked responses (Fig. 1b–d). As controls, we used bitter tastants with unrelated chemical structures such as benzamide (BEN), cycloheximide (CYC), vitamin B1 (thiamine) (B1) and famotidine (FAM) (Fig. 1a). Strong GG-evoked calcium responses were only observed for compounds that share the GG ligand structure. KCl was used as an internal standard to verify cell viability. These results imply that GG neurons are able to recognize this new family of ligands previously known as bitter tastants.

### Intrinsic chemico-physical properties of danger signals are crucial for their olfactory detection by the Grueneberg ganglion

**Fig. 2:**
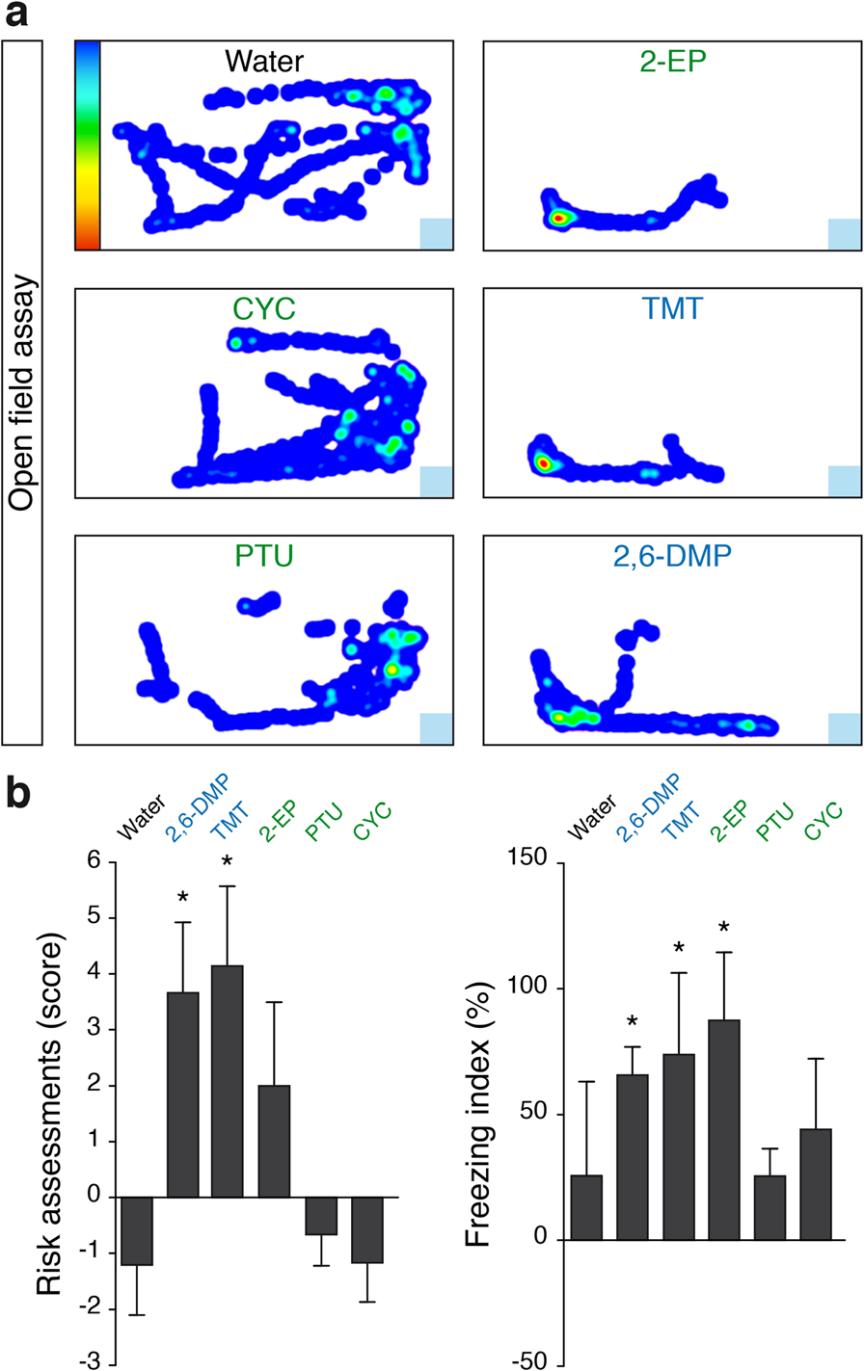
Bitter tastants differentially impact mouse olfactory-related fear behaviours. **a** Representative occupancy plots of mice exposed to control (Water), to bitter tastants 2-EP, CYC and PTU or to GG ligands TMT and 2,6-DMP. *Blue squares* indicate location of tested cues. **b** Scores of risk assessments and index of freezing time obtained with mice exposed to the tested molecules from **a**. Colour-coded map indicates *blue* (low occupancy) to *red* (high occupancy) values in **a**. In **a**, **b**, GG ligands are indicated in *blue* and bitter tastants in *green*. In **b**, data are presented as mean ± standard error of the mean (SEM) of *n* = 6 mice, *P < 0.05 (Wilcoxon paired test)

To test whether these ligands can evoke behavioural responses, we exposed B6 mice to the newly identified GG ligands (2-EP, CYC and PTU) in a set of open field behavioural experiments in which the animals had no physical accessibility to the tested cues [19] and compared the results obtained with the ones recorded in the presence of the known kairomones TMT and 2,6-DMP (Fig. 2a). Tracking the mice, we found that PTU, which is structurally related to known GG ligands, and CYC (unrelated structurally) did not induce aversive reactions as no reduction of their exploration behaviour was observed. On the contrary, 2-EP strongly altered mice behaviour, indicating that this chemical could induce innate aversive reactions by also affecting the sense of smell. We further scored its impact on two stereotypic fear-related behaviours: risk assessment, which is an aversive reaction characterized by a mouse sniffing approach with a low-lying extended body posture followed by body retraction, and freezing time, a fear reaction in which the immobility of the animal with only respiratory movements is observed. We noticed that the presence of 2-EP indeed induced fear as observed by an increase in both of these specific behaviours (Fig. 2b).

**Fig. 3:**
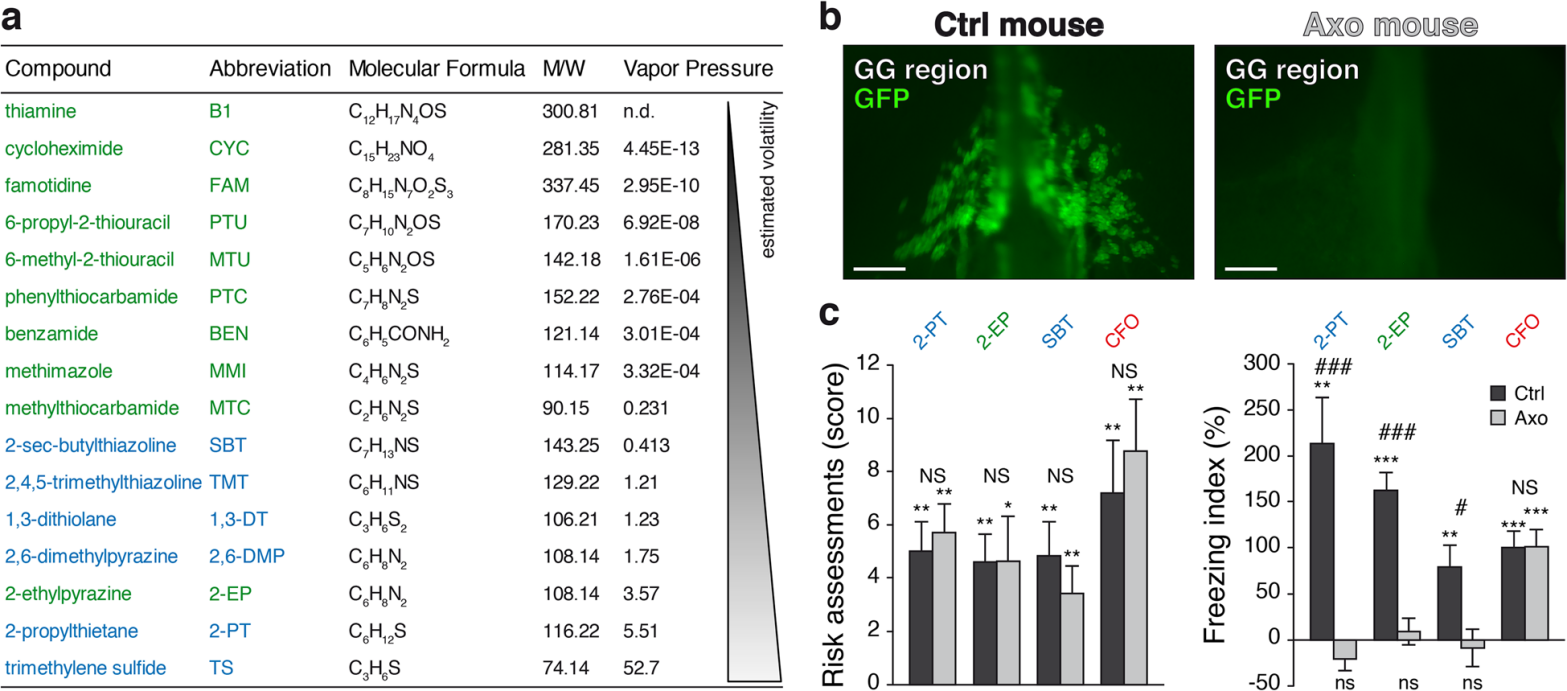
Volatility of danger signals is critical to induce fear behaviours in mice in a GG-dependent manner. **a** GG ligands and bitter tastants sorted according to their volatility. GG ligands are indicated in *blue* and bitter tastants in *green*. The abbreviation, molecular formula and molecular weight (*M/W*) are indicated for each compound. The volatility is estimated from their respective vapour pressures (mmHg at 25 °C) with EPI suite; *n.d.* not determined. **b** OMP-GFP mouse phenotyping. Whole-mount fluorescent view of GG regions from a control (*Ctrl*) and an axotomized (*Axo*) mouse. **c** Comparisons between Ctrl and Axo mice for their score of risk assessments and index of freezing time. Cat fur odour (*CFO*, in *red*) was used as a GG-independent fear inducer. In **b**, scale bars = 0.25 mm. In **c**, data are presented as mean ± SEM of *n* = 6–10 Ctrl mice and *n* = 7–8 Axo mice. For behavioural effects, *P < 0.05; **P < 0.01; ***P < 0.001; *ns* not significant (Wilcoxon paired test). For phenotype comparisons, #*P* < 0.05; ###*P* < 0.001; *NS* not significant (one-tailed Student’s *t* test or Wilcoxon test)

As noted above, we identified both PTU and 2-EP as being structurally similar to known GG ligands; however, only 2-EP elicited a behavioural reaction. From previous experiments, it is known that GG ligands have to be low-weight and volatile molecules [4] to cause aversive reactions in an in vivo situation. Using their vapour pressure, we then calculated, with the Estimation Programs Interface (EPI) suite, the volatility of the putative GG ligands as an estimate of physical/chemical and environmental fate properties of these compounds (Fig. 3a) and found that they are generally poorly volatile. Of those compounds, 2-EP emerges as the only chemical with volatility comparable to the ones of the biologically relevant GG ligands, suggesting that this property may account for its ability to induce a behavioural response.

We next generated axotomized OMP-GFP mice (Axo) where the GG projection bundles have been sectioned, inducing the complete degeneration of the GG [4] to further investigate the specificity with which these compounds acted on this olfactory subsystem. We compared the in vivo effects of 2-EP on OMP-GFP mice (Ctrl versus Axo, Fig. 3b). As an internal control, we used the stoat-derivate kairomone, 2-PT, for its related calculated volatility with 2-EP and also because it evokes avoidance exclusively in the presence of a functional GG [3, 15]. For similar reasons, we also used as a positive control the mouse AP, SBT. 2-EP, 2-PT and SBT were solubilized at 1% in distilled water to minimize activation of unspecific trigeminal pathways [3]. Finally, we used cat fur odour (CFO) as a non-volatile and GG-independent fear inducer [15, 33]. Interestingly, we found that, as for 2-PT, SBT and CFO, the bitter tastant 2-EP was sensed by Axo mice, which still displayed risk assessment behaviour (Fig. 3c). However, the freezing behaviour was abolished in the absence of a functional GG for 2-EP as well as for 2-PT and SBT [3], while no influences were observed for the CFO (Fig. 3c). The presence of a GG (Fig. 3b) is thus necessary for the mouse to display the typical fear behaviour observed in the presence of 2-EP, while the general detection by the other olfactory subsystems is conserved [3, 15, 18]. These results confer fear-inducing properties to the volatile 2-EP and highlight the fundamental role of the volatility of chemical danger signals to allow their olfactory detection by the GG.

### Elements of the bitter transduction cascade are expressed in the mouse Grueneberg ganglion

Taken together, the results described above suggest that elements of the bitter taste signalling might be involved in fear responses conferred by the GG. To explore this possibility further, we searched for the expression of bitter taste receptors in the mouse GG.

**Fig. 4:**
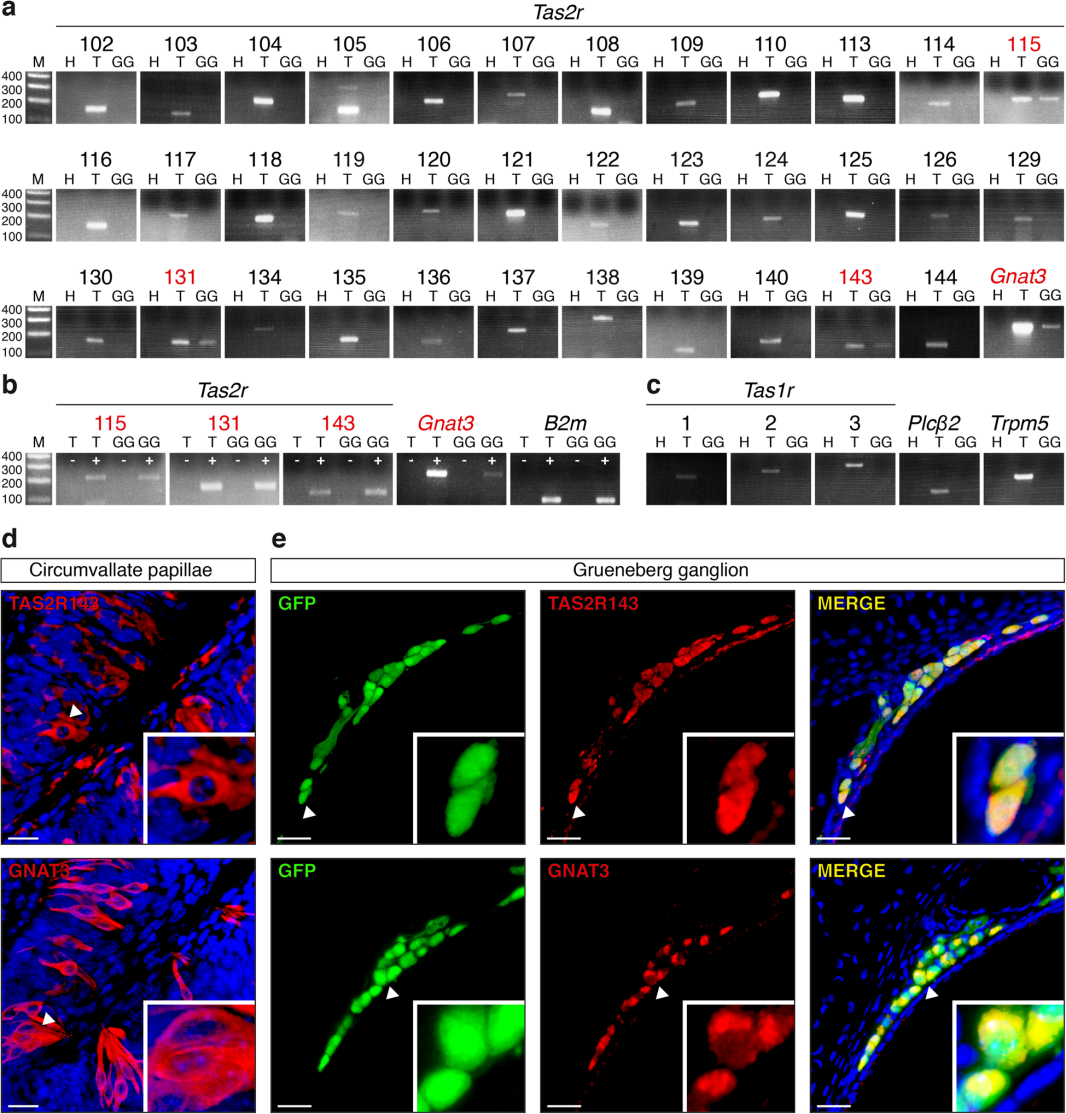
Molecular bitter taste signalling elements are expressed in the mouse Grueneberg ganglion. **a** RT-PCR experiments revealing expression of three bitter taste receptors (*Tas2r115*, *Tas2r131* and *Tas2r143*) and *Gnat3* transcripts in the tongue (*T*) and in the mouse Grueneberg ganglion (*GG*). **b** *Tas2r115*, *Tas2r131, Tas2r143, Gnat3 and B2m* expression in the GG and tongue of mice. + cDNA sample, – negative control omitting the reverse transcription. **c** Transcripts of sweet and umami taste receptors (*Tas1r1, Tas1r2 and Tas1r3)*, phospholipase-C β2 (*Plcβ2*) and of the transient receptor potential channel M5 (*Trpm5*) were found in the tongue, but not in the GG. **d** Immunohistochemistries on mice tongue tissue section (circumvallate papillae) with antibodies against TAS2R143 and GNAT3. **e** Immunohistochemistries on mice GG sections with antibodies against TAS2R143 and GNAT3. GG cells expressing GFP are visible in *green* due to the intrinsic expression of the GFP. In **a**–**c**, *M* is 100-bp ladder and *H* is H_2_O. In **d**, **e**, *white arrowheads* correspond to enlarged views displayed in insets; nuclei are counterstained in *blue* with 4’,6-diamidino-2-phenylindole (DAPI); scale bars are 20 μm

Bitter tastants are recognized by a family of G protein-coupled receptors (GPCRs), the bitter taste receptor family TAS2Rs [27, 30]. These receptors activate the G protein gustducin and the downstream signalling elements phospholipase C-beta 2 (PLCβ2) and the transient receptor potential channel M5 (TRPM5) [34–37]. We searched for the expression of TAS2Rs and their downstream signalling elements using reverse transcription-polymerase chain reaction (RT-PCR) (Additional file 1: Table S1) on laser-microdissected mouse GG neurons. Thirty-five TAS2Rs have been previously identified in mice [38], and we found three different transcripts in the GG of adult mice, *Tas2r115*, *Tas2r131* and *Tas2r143*. We also found their associated alpha G protein gustducin (*Gnat3*) [35] (Fig. 4a). Control experiments showed the absence of amplicons in *Tas2r115*, *Tas2r131*, *Tas2r143* and *Gnat3* when the RT step was omitted. The beta-2-microglobulin (*B2m*) was used as an internal standard control (Fig. 4b). We did not find transcripts for the sweet and umami receptors *Tas1r1*, *Tas1r2*, *Tas1r3* [39] in GG neurons (Fig. 4c). Furthermore, we were unable to detect the other elements of the bitter transduction cascade, *Plcβ2* and *Trpm5* (Fig. 4c), suggesting that alternative signalling pathways might be used instead in mouse GG neurons [15, 20, 40].

To verify further the expression of the taste receptors in the GG, we raised antibodies against the TAS2R115, TAS2R131 and TAS2R143 proteins. We first tested the specificity of these antibodies by staining mouse tongue tissue slices, focusing on the taste receptor cells present in the circumvallate papillae [30]. Among the three antibodies we developed, only the ones raised against TAS2R131 and TAS2R143 were functional and specifically stained a subset of taste receptor cells (Fig. 4d, Additional file 2: Figure S1). We then looked for the expression of these two TAS2Rs in GG neurons and detected their expression in most GG neurons (Fig. 4e, Additional file 2: Figure S1). We also verified by immunohistochemistry the presence of the α-subunit of gustducin (GNAT3) and found it to also be expressed in the GG of the mouse (Fig. 4e). These results demonstrate that TAS2R131, TAS2R143 and the α-subunit of gustducin are expressed in the mouse GG.

### Mouse alarm pheromone and predator-derived kairomones activate STC-1 cells

**Fig. 5:**
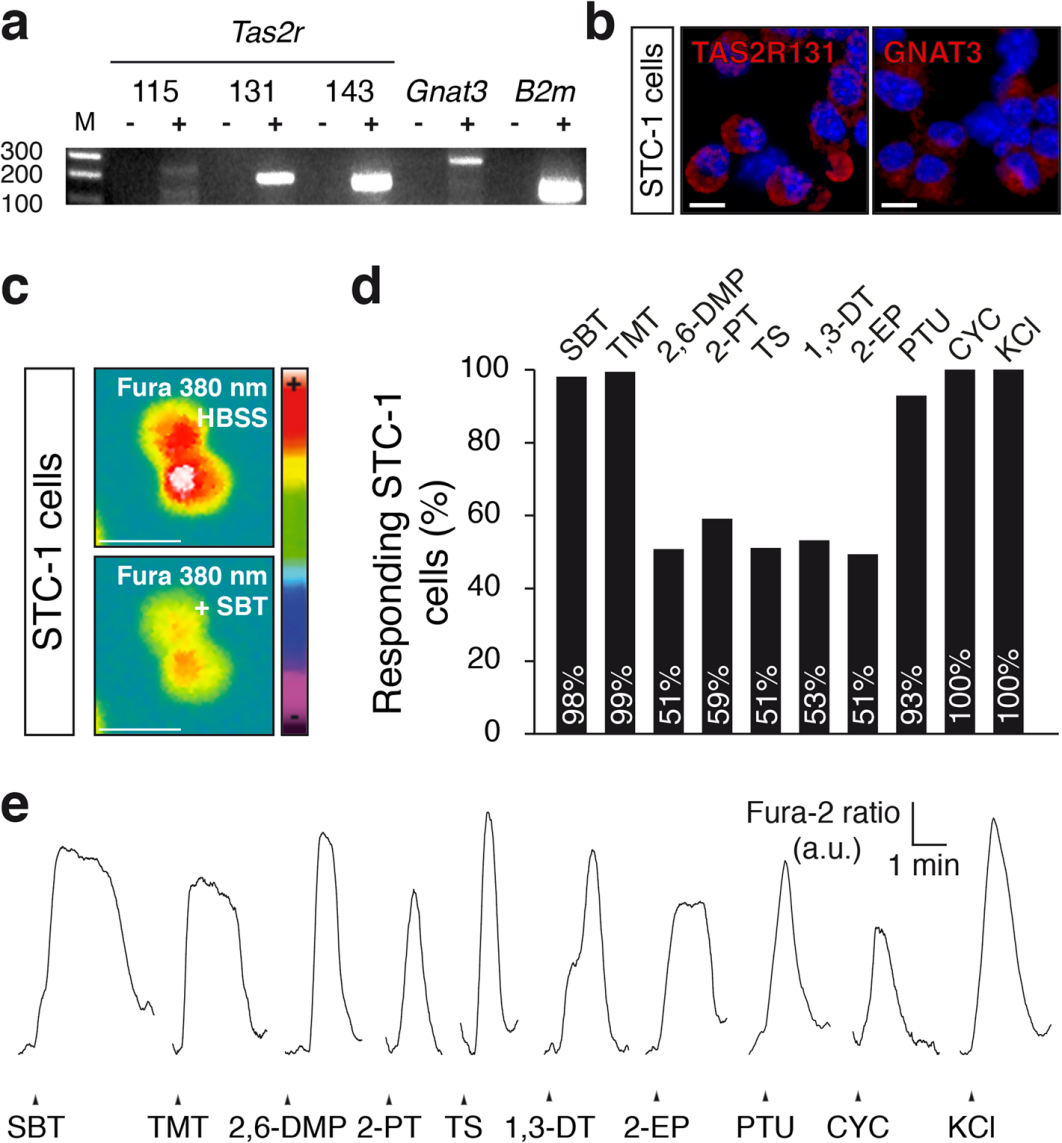
STC-1 cells respond to GG ligands. **a** RT-PCR experiments revealing expression of the three *Tas2r* transcripts, *Tas2r115, Tas2r131* and *Tas2r143*, in STC-1 cells. Expression of *Gnat3* and of the positive control *B2m* were also confirmed. + cDNA sample, – negative control omitting reverse transcription step. **b** Immunocytochemistries on STC-1 cells with antibodies against the proteins TAS2R131 and GNAT3. **c** Fura-2 AM uptake in STC-1 cells measured at 380 nm in a colour-coded map in basal conditions with Hank’s Balanced Salt Solution (*HBSS*) and during activation by the alarm pheromone 2-*sec*-butylthiazoline (*SBT*). **d** Bar graph representing the response rate induced in STC-1 by GG ligands and bitter tastants tested in **e**. SBT (299/305); 2,4,5-trimethylthiazoline, *TMT* (488/491); 2,6-dimethylpyrazine, *2,6-DMP* (35/69); 2-propylthietane, *2-PT* (127/215); trimethylene sulphide, *TS* (50/98); 1,3-dithiolane, *1,3-DT* (52/98); 2-ethylpyrazine, *2-EP* (35/69); 6-propyl-2-thiouracil, *PTU* (78/84); cycloheximide, *CYC* (163/163). **e** Representative calcium transients induced by the alarm pheromone SBT and by the kairomones TMT, 2,6-DMP, 2-PT, TS and 1,3-DT respectively and by the bitter tastants 2-EP, PTU, CYC at (100 μM) and by a control pulse of KCl (10 mM). In **b**, **c**, scale bars = 15 μm. In **b**, nuclei are counterstained in *blue* with DAPI. In **d**, *n*/*x* = number of cells responding/number of cells tested and viable. In **e**, fluorescence intensity Fura-2 ratio = F340/F380 is indicated by arbitrary units (*a.u.*); time is indicated by a horizontal bar

We next took advantage of the STC-1 mouse enteroendocrine tumoural cell line, which is known to express the TAS2Rs and has been reported previously to be activated by bitter tastants [34, 41, 42], to verify whether the newly identified GG ligands, the mouse AP and predator-derived kairomones were indeed able to activate these receptors. We first verified that the three TAS2Rs and the GNAT3 subunit present in the GG were found in these cells at the messenger RNA (mRNA) and the protein levels (Fig. 5a, b). We then perfused the STC-1 cells with ligands of the GG (SBT, TMT, 2,6-DMP, 2-PT, TS, 1,3-DT) and bitter tastants (2-EP, PTU, CYC) during calcium imaging experiments where the cells were first loaded with the calcium dye Fura-2 AM (Fig. 5c). As expected, the mouse AP, SBT, as well as the kairomones and the bitter tastants tested induced reversible calcium transients in STC-1 cells (Fig. 5c–e). KCl was used as a viability test. These results suggest that GG ligands are able to activate TAS2R-expressing cells.

### TAS2R143 identified in the mouse Grueneberg ganglion responds to alarm pheromone and predator-derived kairomones

While bitter tastants are able to activate TAS2R-expressing STC-1 cells, given that these receptors can recognize multiple bitter tastants [27, 29], we needed to determine the specificity of this interaction. To this aim, we performed ligand-receptor identification in a heterologous system for single receptor expression [43, 44].

**Fig. 6:**
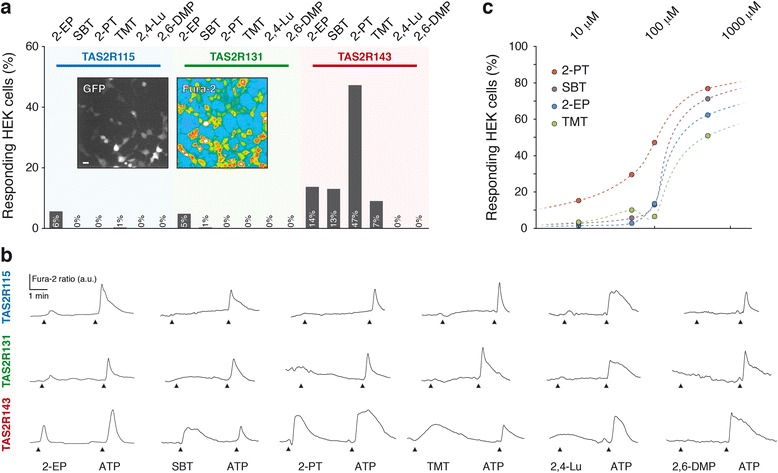
TAS2R143 is a narrowly tuned GG ligand receptor. **a** Bar graph representing the response rate induced in HEK cells transfected respectively with TAS2R115, TAS2R131 and TAS2R143. Superimposed insets display a representative successful transfection due to GFP expression and Fura-2 AM uptake measured at 380 nm in a colour-coded map. For TAS2R115, 2-ethylpyrazine, *2-EP* (8/144); 2-*sec*-butylthiazoline, *SBT* (0/310); 2-propylthietane, *2-PT* (0/104); 2,4,5-trimethylthiazoline, *TMT* (2/205); 2,4-lutidine, *2,4-Lu* (0/112); 2,6-dimethylpyrazine, *2,6-DMP* (0/65). For TAS2R131, 2-EP (7/147), SBT (2/203), 2-PT (0/333), TMT (0/248), 2,4-Lu (0/183), 2,6-DMP (0/171). For TAS2R143, 2-EP (20/146), SBT (45/345), 2-PT (112/237), TMT (9/236), 2,4-Lu (0/58), 2,6-DMP (0/84). **b** Representative calcium transients induced by the bitter tastant 2-EP as well as by the alarm pheromone SBT and by the kairomones 2-PT, TMT, 2,4-Lu and 2,6-DMP at (100 μM) and by a control pulse of ATP (100 μM) on HEK cells transfected with TAS2R143, TAS2R131 and TAS2R115 respectively. **c** Detail of the pseudo dose response obtained for TAS2R143 and its responding ligands from **a**. In **a**, scale bars = 15 μm; *n*/*x* = number of cells responding/number of cells tested and viable. In **b**, fluorescence intensity Fura-2 ratio = F340/F380 is indicated by arbitrary units (*a.u.*); time is indicated by a horizontal bar. In **c**, the tested concentrations are indicated by coloured dots (2-PT in *red*, SBT in *purple*, 2-EP in *blue* and TMT in *green*); as a minimum, 58 viable cells were used for each cue and concentration tested

We expressed TAS2Rs in human embryonic kidney 293 (HEK) cells and challenged the system with 2-EP, which showed activity in our behavioural analysis and cell activation assay as well as with the only identified, so far, mouse AP (SBT), and the representative kairomones emitted by the main mice predator families, such as TMT from the Canidae, 2-PT from the Mustelidae and from the Felidae, 2,4-lutidine (2,4-Lu) and 2,6-DMP. At a concentration of 100 μM [3, 19], of all the compounds tested only 2-EP was able to initiate small calcium increases in around 5% of the cells transfected with the single receptors TAS2R115 and TAS2R131 (Fig. 6a, b). This might be due to insufficient membrane expression of the receptors or to the inability of the tested ligands to activate them. On the other hand, we found that HEK cells transfected with TAS2R143 responded respectively to 2-EP (14%), SBT (13%), 2-PT (47%) and TMT (7%) (Fig. 6a, b) with thus a special tuning towards 2-PT as 15% of the cells still responded at 10 μM (Fig. 6c). Interestingly, at 500 μM, TAS2R143-transfected cells responded as well to the mouse AP SBT (71%) as to the predator-derived kairomones TMT (51%) and 2-PT (77%). As a control, we verified that the three receptors were not endogenously expressed in HEK cells and that, when cells were transfected with an empty vector and the GFP reporter gene, they did not respond to the tested ligands (Additional file 3: Figure S2)*.* In summary, these experiments demonstrate the ability of mouse chemical danger cues to activate the bitter receptor TAS2R143 at concentrations that might be encountered in the wild [1, 3, 19].

## Discussion

Mice have evolved different sensory systems to perceive environmental dangers and to allow their survival [1]. The Grueneberg ganglion (GG) is an olfactory subsystem that detects a large number of volatile compounds displaying heterocyclic structures, including analogues of pyrazines, pyridines, thiazoles, thiazolines or thietanes [3, 19, 45, 46]. Here we have been able to activate the GG with a specific category of bitter tastants composed mostly of analogues of heterocyclic compounds. A few of the compounds predicted to act as activators nonetheless did not show activity in behavioural and imaging assays. In the case of methimazole, this may be due to the absence of its specific TAS2R [29] in the GG or to its known olfactory toxic effect [47–49]. Large molecules, such as vitamin B1 or famotidine, although they bear a thiazole ring, a moiety found in GG ligands, failed to activate GG neurons, confirming that ligand recognition is influenced by the steric bulk [45, 46]. Furthermore, the volatility of the compounds was shown to be pertinent for behavioural experiments: The bitter tastant PTU, which strongly activated GG neurons in tissue slices during calcium imaging experiments but possesses poor volatility, failed to induce a behavioural response. On the other hand, the volatile bitter tastant 2-EP, which also induced robust calcium responses in GG neurons, was able to induce fear behaviours in the presence of a functional GG. Interestingly, we notice that analogues of 2-EP were previously found in the urine of the mountain lion [19], a mouse predator. 2-EP might therefore act as a predator-derived kairomone. These results illustrate the critical importance of the volatility of the danger signals to allow their olfactory screening by mice and the generation of an appropriate response to improve their fitness. Hence, cues with similar chemical structures could bring the same information (danger) and induce the same behaviour (fear) when they are detected by the same receptors, but their localization in morphologically separate sensory systems might allow detection according to their physical accessibility (volatility) and natural encounter probability (tastants or odourants).

Numerous families of sensory GPCRs have been reported to be co-expressed in the mouse GG, such as a vomeronasal type 2 receptor (V2r83), an olfactory receptor (MOR256-17) and multiple trace amine-associated receptors (TAAR2/4/5/6/7), in addition to the guanylyl cyclase type G (GC-G) [20–23, 50, 51]. Here, we demonstrate that mouse GG neurons express three members of the TAS2R family, membrane receptors previously identified as bitter tastant receptors [30]. This implies that the GG might take advantage of several receptors to detect multiple families of chemical signals in the same neuron [19]. Indeed, and in parallel to our manuscript submission process, a recent study demonstrated that GC-G is significantly implicated in the perception of the AP, SBT, via the GG [24]. Genetic deletion of GC-G reduced the responsiveness to SBT in GG neurons and attenuated fear-related behaviours in mice. Given that the attenuation of the fear-related behaviours in these mice was partial, additional receptors, such as the bitter taste receptors, could be implicated in detecting the AP SBT [24]. On the other hand, detection of TMT by GG neurons does not rely on cGMP signalling [15] and thus is independent of GC-G [24]. Thereby, TAS2R143 might be a good candidate for the detection of this fox-derived kairomone by the mouse GG. In summary, we can thus speculate that GC-G and TAS2Rs as well as other receptors could collaborate to optimize the molecular range of detection by overlapping their specificity. Remarkably, TAS2Rs are not only limited to the oral cavity. They have been found in the human thyroid [52], the murine enteroendocrine cells of the gut and pancreas [42], the heart [53], the nasal cavity and more specifically in the solitary chemosensory cells (SCCs) [54]. Interestingly, SCCs, distributed throughout the nasal cavity, are able to recognize irritants, inducing specific repulsive reactions like sneezing and apnea [54]. This organo-specific expression implies a vital protective role. We now have found TAS2Rs expression in the mouse GG, where they participate in the detection of chemical molecules signalling danger, adding an olfactory warning function for this receptor family.

The proposed bitter taste signalling pathway includes additional specific molecular elements, such as PLCβ2 and TRPM5 [55], that we did not find in mouse GG neurons. Thus, these observations suggest that the GG might use a different second messenger and different effectors. The cyclic nucleotide-gated channel type A3 (CNGA3) is indeed expressed both in the GG [20, 23] and interestingly also in the taste receptor cells, referred to as CNGgust [56] and might be involved both in taste signal transduction [56, 57] and in odourant-evoked GG responses [50, 58]. Moreover, intracellular calcium stock from the endoplasmic reticulum could also be solicited, as this sarco/endoplasmic reticulum Ca^2+^-ATPase (SERCA)-dependent pathway occurs both in taste signalling and in the GG [4, 59]. The expression of multiple receptor families as well as convergent and/or parallel signalling profiles in the same GG neuron, similar to what has been described in taste receptor cells [60, 61], could be considered as an evolutionary attribute that expands the neuronal tuning profile to a given category of odourants [45, 62] such as the chemical danger cues.

## Conclusions

In the wild, preys detect the presence of their predators and avoid being eaten through the detection of predator scents and of their stressed or injured conspecifics via alarm pheromone sensing [4, 63–66]. Here, we found three TAS2Rs expressed in the mouse GG neurons that might be implicated in this sensory deciphering. We were not able to determine the molecular receptive range of TAS2R115 and TAS2R131. However, we show that, in addition to the bitter tastant 2-EP, TAS2R143 is activated by the mouse alarm pheromone SBT and by the kairomone TMT, as well as by 2-PT, a stoat-emitted kairomone. As GG neurons express multiple signalling elements that could act as parallel or convergent transduction signals, future studies using knockout mice for *Tas2r143* should establish the biological relevance of this bitter receptor for the detection of volatile danger cues mediated by the GG and for determining how the integration of the multiple environmental signals takes place in this olfactory subsystem.

## Methods

### Animals

Male and female B6 (C57BL/6 J; Janvier Labs; 2–5 months) and OMP-GFP (olfactory marker protein-green fluorescent protein; a kind gift from I. Rodriguez, University of Geneva, Switzerland; 0–5 months) gene-targeted mice were used. In these mice, the GFP is expressed instead of OMP in all mature olfactory neurons under the OMP promoter [32]. Mice were maintained in a normal light cycle (12:12). Animal care was in accordance with Swiss legislation and approved by the EXPANIM committee of the Lemanic Animal Facility Network and the veterinary authority of the Canton de Vaud (SCAV).

### Behavioural experiments

For general olfactory investigations, B6 male mice were used and were accustomed to the test arena (a closed Plexiglas box of dimensions 45 × 25 × 19 cm) in the presence of 1 mg of water in a 2-ml Eppendorf tube, to minimize environmental stress. During the experiments, mice were placed in the centre of the arena and exposed to 1 mg of olfactory cues or bitter tastants (from Sigma-Aldrich, Contech or house-made [3]) placed in a 2-ml Eppendorf tube in the corner of the cage. Before each tested cue session, a control session was performed with only water and used to compare occupancy plots, to score the risk assessment (sniffing approach with low-lying extended body posture followed by body retraction) and to calculate as a percentage the freezing index (animal immobility with only respiratory movements) [3, 19]. These behaviours were manually scored during the first 3 min of the behavioural assay with the Any-maze software (version 4.99) [19].

For GG-dependent investigations, GG axotomized OMP-GFP mice were generated according to previous reports [3, 4]. Briefly, under deep anaesthesia, projection bundles of GG neurons were sectioned, a procedure that allowed the complete and rapid degeneration of the ganglion [7]. A similar procedure was performed with a slight and superficial cut of the rhinarium to generate a mouse control sham-operated group (Ctrl mouse) with an intact GG. Similar behavioural experiments as described above were performed at least 30 days after the lesion. We delivered 100 μl of 1% water-diluted tested cues on odourless cotton pads. CFO was obtained by rubbing the neck region of three domestic adult cats (either gender) with cotton pads. At the end of the behavioural assays, degeneration of the GG was phenotyped using a whole-mount preparation under a fluorescence stereomicroscope (MZ16FA/M165FC; Leica). Absence of GFP-positive neurons in the GG region was used as a criterion to consider the axotomized mouse group (Axo mouse) [3, 4].

### Calcium imaging experiments on Grueneberg ganglion neurons

As described in Brechbühl et al. [3], pups (post natal days P0–P7) were killed by decapitation, and the GG regions were removed and carefully placed in artificial cerebrospinal fluid (ACSF), consisting of 118 mM NaCl, 25 mM NaHCO_3_, 10 mM d-glucose, 2 mM KCl, 2 mM MgCl_2_, 1.2 mM NaH_2_PO_4_, 2 mM CaCl_2_ (pH 7.4), and gassed with oxycarbon (95% O_2_, 5% CO_2_). They were embedded in 5% agar prepared in phosphate-buffered saline (PBS), and acute 80-μm tissue slices were then processed with the microtome. The tissue slices were then loaded with a solution of 0.1% Pluronic (F-127, Invitrogen) and 5 μM Fura-2 AM (TEFLabs) in ACSF for 45 min. The slices were then placed in the bath chamber (RC-26, Warner Instruments). Calcium variations were observed with an inverted microscope (Axiovert 135, Zeiss) using a 63× objective and a CoolSNAP HQ (Photometrics) camera. MetaFluor (Visitron) software was used to monitor calcium variations.

### Chemostimulation of Grueneberg ganglion neurons

Cues were purchased from Sigma-Aldrich and used for stimulation of acute tissue slices. They were dissolved in dimethyl sulphoxide (DMSO) and delivered in ACSF. The osmolarity of solutions was between 285 and 300 Osm/L. We perfused these molecules at 1 mM, a concentration generally used in the characterization of taste response experiments [41, 42]. Responses induced by cues were standardized by comparison of the intracellular calcium increase with a short pulse of KCl 20 mM. The baseline activity of the cells was observed during ACSF perfusion and corresponded to 5% of a KCl stimulation. Intracellular calcium increases greater than twice the increase of baseline activity (10%) of a KCl response were considered as responses [45].

### Laser capture dissection and RNA isolation

Male adult C57BL/6 J and OMP-GFP mice were euthanized with CO_2_, and organs of interest were rapidly dissected and placed in OCT Compound (Tissue-Tek). The OCT blocks containing the tissue were then cooled on dry ice for 5 min. RNAse free MembraneSlides (50102, Molecular Machines & Industries) were sterilized under ultraviolet (UV) light for 15 min and coated with poly-l-lysine (Sigma) for 30 min. Slices 14 μm thick were processed. Tissue sections were then dehydrated and placed consecutively into 75% ethanol (EtOH) for 1 min, diethylpyrocarbonate (DEPC)-treated water for 1 min, 75% EtOH for 1 min, 95% EtOH for 1 min, 100% EtOH for 1 min and 100% xylene for 3 min and then air dried for 3 min. Regions of interest were then finely dissected using a laser-capture microdissection system composed of an inverted microscope (IX71, Olympus) and a Laser Microdissection System (CellCut, Molecular Machines & Industries). Diffuser caps (50202, Molecular Machines & Industries) were used to retrieve microdissected tissues. Five slices were retrieved per diffusion cap. RNA was extracted from microdissected tissues using an RNA Isolation kit (Arcturus PicoPure RNA Isolation Kit, Life Technologies) following the manufacturer’s instructions. Briefly, microdissected tissues were placed in 50 μl extraction buffer (XB) for 30 min at 42 °C. After incubation, the sample was centrifuged and loaded onto a spin column. To avoid genomic DNA contamination, an additional digestion step using DNAse I (RNase-Free DNase Set, Qiagen) was performed. The sample was then washed several times, and the total RNA was eluted in a total volume of 30 μl.

### Reverse transcription-polymerase chain reaction (RT-PCR)

The complementary DNA (cDNA) synthesis kit (PrimeScript First Strand cDNA Synthesis Kit, Takara) was used according to the manufacturer’s instructions. Briefly, cDNA was synthesized using random hexamers, and samples were eluted in a total volume of 20 μl. PCR experiments were performed using cDNA corresponding to 5 ng of the reverse transcripted RNA and 400 nM primers. Each primer was designed using the website Primer3Plus [67]; the primers are listed in Additional file 1: Table S1. Amplification was performed with 1.25 units of GoTaq DNA Polymerase (Promega) in a thermocycler (peqSTAR) at 95 °C, 30 s, 58–62 °C, 30 s and 72 °C, 30 sec for 35 cycles. Amplification products were visualized with ethidium bromide on a 3% electrophoresis gel. Negative controls were performed omitting the reverse transcription step or with H_2_O. A 100-bp ladder (Promega) was used to verify the size of the amplification products.

### Immunohistochemistry on floating slices

Immunohistochemistry experiments were adapted from Brechbühl et al. [51]. Briefly, male and female adult C57BL/6 J and OMP-GFP mice were euthanized with CO_2_, and organs of interest were dissected and placed in PBS (138 mM NaCl, 2.7 mM KCl, 1.76 mM KH_2_PO_4_ and 10 mM Na_2_HPO_4_, pH 7.4). The tips of the noses and mice tongues were then fixed in 4% paraformaldehyde (PFA 4%, in PBS pH 7.4; 158127, Sigma) at 4 °C for 3 h. Tissues were embedded in 5% agar (A7002, Sigma) prepared in PBS. Acute 80- to 100-μm tissue slices were then processed with a microtome (VT1200S, Leica). The tissue slices were placed in a blocking solution of 10% normal goat serum (NGS, Jackson ImmunoResearch) and 0.5% Triton X-100 (Fluka) in PBS overnight at room temperature. Tissue slices were then placed in a solution containing the primary antibody, 5% NGS and 0.25% Triton-X-100 in PBS for 16 h at room temperature. Tissue slices were rinsed three times with a solution of 2% NGS in PBS 5 min at room temperature. The secondary antibodies were applied to the tissue slices in a solution of 2% NGS in PBS 1.5 h at room temperature. Tissue slices were finally washed three times in a decreasing concentration solution of NGS in PBS (2% NGS, 1% NGS and PBS) 5 min at room temperature and mounted in Vectashield with 4’,6-diamidino-2-phenylindole (DAPI) (H-1200, Vector Labs) mounting medium. Control experiments omitting the secondary antibody were performed for each primary antibody. The primary antibodies used against the TAS2Rs were against TAS2R115, TAS2R131 and TAS2R143. They were raised by GenScript (Rabbit, 1:100, GenScript USA Inc.) against the intracellular part of the proteins to produce affinity-purified polyclonal antibodies. The antibody against the GNAT3 protein was commercially obtained (Gustducin, sc-395, RRID: AB_10177605, Rabbit, 1:250, Santa Cruz Biotechnology). The secondary antibodies used were coupled to Cy3 (Cy3-conjugated AffiniPure anti-Rabbit, 111-165-144, RRID: AB_2338006, Goat, 1:250, Jackson ImmunoResearch). Image acquisitions were performed by confocal microscopy (SP5, Leica) using a 40× objective. Image optimization was performed with Imaris 7.0 (Bitplane).

### Cell culture

The STC-1 cells, a kind gift from D. Hanahan (Ecole Polytechnique Fédérale de Lausanne, Switzerland), are an enteroendocrine cell line originated from a double-transgenic mouse tumour [42, 68]. Cells were grown in Dulbecco’s modified Eagle’s medium (DMEM) supplemented with 10% foetal bovine serum (FBS) and 100 U/ml penicillin, and 100 mg/L streptomycin and incubated in a 5% CO_2_ atmosphere at 37 °C. Cells were used between passages 30 and 50. HEK cells (ATCC CRL-1573) were grown in DMEM supplemented with 10% FBS and 100 U/ml penicillin, and 100 mg/L streptomycin and incubated in 5% CO_2_ at 37 °C. Cells were used between passages 10 and 30.

### Transfected HEK cells

#### Plasmid construction

Protocols were adapted from the procedure in Ueda et al., 2003 [44]. Briefly, mouse bitter taste receptors TAS2R115, TAS2R131 and TAS2R143 were cloned into a pcDNA3.1 (+) mammalian expression vector (GenScript) and tagged in its N-terminal position with the first 39 amino acids of the bovine rhodopsin receptor, facilitating the cell surface expression. The G α-subunit was designed by constructing a chimera of the human Gα16 where the C-terminal 44 amino acids of the mouse gustducin G protein α-subunit replaced those of the Gα16. This chimera was cloned into a pcDNA3.1 (+) mammalian expression vector (GenScript).

#### Cell culture and transfection

Human embryonic kidney 293 (HEK) cells were grown in DMEM, supplemented with 10% FBS and 100 U/ml gentamycin and incubated in a 5% CO_2_ atmosphere at 37 °C. Cells were transfected via calcium phosphate transfection method [69] and were cotransfected with the Gα16, the TAS2R and with a GFP reporter plasmid to validate the transfection and to estimate the transfection rate. Cells were then processed for calcium imaging or immunocytochemistry after 48 h of transfection.

### Chemostimulation of cells

Cues were purchased from Sigma-Aldrich, Alfa Aesar and Contech at the highest available purity or synthesized in-house [3, 19]. Molecules were delivered in Hank’s Balanced Salt Solution with calcium, magnesium (HBSS, Sigma, H8264) or Ringer [70]. The osmolarity of solutions was between 285 and 300 Osm/L. STC-1 cells are known to respond to bitter tastants in a range from 50 μM to 10 mM [42]. If not otherwise mentioned, ligands were perfused at 100 μM, an intermediate physiological concentration in calcium imaging experiments on STC-1 and on native or transfected HEK cells.

### Immunocytochemistry on STC-1 and HEK cells

Immunocytochemistry experiments were adapted from Brechbühl et al. [51]. Briefly, after fixation and blocking phases, the cells were processed for indirect immunostaining and mounted in Vectashield with DAPI (H-1200, Vector Labs) mounting medium. The already-mentioned primary antibodies against TAS2R115, TAS2R131, TAS2R143 and GNAT3 were used on both STC-1 and HEK cells. The secondary antibodies used were coupled to Cy3 (Cy3-conjugated AffiniPure anti-Rabbit, 111-165-144, RRID: AB_2338006, Goat, 1:250, Jackson ImmunoResearch). Image acquisitions were performed by confocal microscopy (SP5, Leica) and developed with Imaris 7.0 (Bitplane).

### Statistical analyses

Values are expressed as mean ± standard error of the mean (SEM). For the behavioural assays, the Wilcoxon paired test was used for comparisons. Significance levels are indicated as follows: *P < 0.05, **P < 0.01, ***P < 0.001, ns: not significant. For comparisons between phenotype groups (Ctrl versus Axo mouse), the one-tailed Student’s *t* test or Wilcoxon test was used, and the significance levels are indicated as follows: #*P* < 0.05, ###*P* < 0.001, NS: not significant. *n* = number of cells if not otherwise mentioned.

## Additional files

Additional file 1: Table S1. Primers used in RT-PCR. (DOCX 37 kb)

Additional file 2: Figure S1. Grueneberg ganglion neurons express TAS2R131. (**a**) Immunohistochemistries on mice tongue tissue section (circumvallate papillae) with antibodies against TAS2R131 with and without its competing peptide to verify the staining selectivity. (**b**) Immunohistochemistries on mice GG sections with antibodies against TAS2R131 with and without its competing peptide to verify the staining selectivity. GG cells expressing GFP are visible in *green* due to the intrinsic expression of the GFP. *White arrowheads* correspond to enlarged views displayed in insets; nuclei are counterstained in *blue* with DAPI; scale bars are 20 μm. (TIF 4156 kb)

Additional file 3: Figure S2. HEK cells are not activated by Grueneberg ganglion ligands. (**a**) Immunocytochemistry experiments revealing the absence of expression of TAS2R131 and GNAT3 in HEK cells. (**b**) Representative intracellular calcium responses on HEK cells following perfusion of the alarm pheromone 2-*sec*-butylthiazoline (*SBT*) and of a mix of kairomones (TMT, 2-PT, 2,6-DMP and 2,4-Lu) at 100 μM. ATP was perfused as an internal control at 100 μM. In (**a**), *white arrowheads* correspond to close-up view of single HEK cell (insets). Scale bars are 30 μm. Nuclei are counterstained in *blue* with DAPI. In (**b**), fluorescence intensity Fura-2 ratio = F340/F380 is indicated by arbitrary units (*a.u.*); time is indicated by a horizontal bar. (TIF 1625 kb)

## Abbreviations

1,3-DT: 1,3-dithiolane
2,4-Lu: 2,4 lutidine
2,6-DMP: 2,6-dimethylpyrazine
2-EP: 2-ethylpyrazine
2-PT: 2-propylthietane
AP: alarm pheromone
Axo: axotomized mice
B1: vitamin B1 (thiamine)
*B2m*: beta-2-microglobulin
BEN: benzamide
CFO: cat fur odour
CNGA3: cyclic nucleotide-gated channel type A3
CYC: cycloheximide
FAM: famotidine
GC-G: guanylyl cyclase-G receptor
GG: Grueneberg ganglion
GNAT3: G protein α-gustducin
GPCR: G protein-coupled receptor
HEK: human embryonic kidney (cells) 293
MMI: methimazole
MTC: methylthiocarbamide
MTU: 6-methyl-2-thiouracil
OMP-GFP: olfactory marker protein-green fluorescent protein
PLCβ2: phospholipase C-beta 2
PTC: phenylthiocarbamide
PTU: 6-propyl-2-thiouracil
SBT: 2-*sec*-butylthiazoline
SCC: solitary chemosensory cell
TAS2R: bitter taste receptor
TMT: 2,4,5-trimethylthiazoline
TRPM5: transient receptor potential channel M5
TS: trimethylene sulphide
