# Supplementary material for: Alarm pheromone and kairomone detection via bitter taste receptors in the mouse Grueneberg ganglion
Source: BMC Biol. 2018 Jan 18;16:12. doi: 10.1186/s12915-017-0479-y (PMC5774136; doi:10.1186/s12915-017-0479-y)
Supplement: Supplementary file 1 — Primers used in RT-PCR. (DOCX 37 kb) [file 12915_2017_479_MOESM1_ESM.docx]

Additional file 1: Table S1. Primers used in RT-PCR.

| **Gene** | **Primer Forward Sequence 5' 🡪 3'** | **Primer Reverse Sequence 5' 🡪 3'** | **Annealing**  **Temperature (C°)** | **Fragment**  **Size (bp)** |
| --- | --- | --- | --- | --- |
| *Tas1r1* | AGGCCACCTAGAGATGCAGA | CCGCACTATGACTTCCACCT | 62 | 232 |
| *Tas1r2* | CCTAACGAGACCAGCCTGAG | TCGTCGAAGAAGAGCTGGTT | 60 | 272 |
| *Tas1r3* | AAAGGGCTTTCATTCCTGCT | CTAGGCAGATCAGGCCAAAG | 59 | 311 |
| *Tas2r102* | AATGTGGACCGAGCTGATCT | CCTTGGTGCTAGGGTCTCTG | 60 | 158 |
| *Tas2r103* | CCATCTCCAGGACTGCTTTT | GCATGTTGCAAACCAGACAC | 59 | 152 |
| *Tas2r104* | TGAGTGCACTGGAAAGCATC | AGACTGCTCGGCATAAGCAT | 60 | 238 |
| *Tas2r105* | GGGACCTGGAGATATGCTGA | CGATGAGAAGGAAGCCAGTC | 59 | 161 |
| *Tas2r106* | CACCAGCCTCAACCTCTTCT | GGTAAACCTGCCAGGAAACA | 60 | 200 |
| *Tas2r107* | TCCACCAGCGGAAAAATTAC | TGGAAAGGCCTACAAAATGC | 58 | 243 |
| *Tas2r108* | TGGATGCAAACAGTCTCTGG | GGTGAGGGCTGAAATCAGAA | 59 | 158 |
| *Tas2r109* | TAAAATCCTCACCGCTTTGG | CTGAGGCATGTAGCAAACCA | 58 | 177 |
| *Tas2r110* | TGCAAACAGGGTTCTCCTTC | TATCTTTGGATCGGCACCTC | 58 | 228 |
| *Tas2r113* | GCCAAAGGATGCAGAGACAT | CATGACAAATGGGTGAGCAG | 59 | 201 |
| *Tas2r114* | CTGGGCATTGTAGGGAACAC | CAGGAGACAGCAACTGTGGA | 60 | 181 |
| *Tas2r115* | CTGTGGTTGCCTTCCTCCTA | TCTCACGCTTGCACCAATAC | 60 | 222 |
| *Tas2r116* | GGTTTGGAGTGCTCTGCTTC | ATTCTTTGTGCCTGCACCTC | 60 | 167 |
| *Tas2r117* | TGCTACATGCCTCACTGTCC | CTGCCTGTGACACTTTGCAT | 60 | 238 |
| *Tas2r118* | CCTGGGCATCTCACATTTCT | GAGGAAGATGGGGTGAGTGA | 59 | 196 |
| *Tas2r119* | TCCACTTTGGAAGCAGAACC | TGAGCAGGATGTCTTGTTGG | 59 | 236 |
| *Tas2r120* | TGCATATCTTGGGATGGTGA | CCATGGTGCTCTGATCTTGA | 58 | 245 |
| *Tas2r121* | CAGCACTAAAGCCCATGTGA | ATCACCCAAAGACTGGCTTG | 59 | 222 |
| *Tas2r122* | ATTGTGGCAAGCTCCATTCT | CCTCCACAATGACACACCAG | 59 | 164 |
| *Tas2r123* | CGGGAATCTTAGGACATGGA | ATGTGGGCACAGCATGAATA | 58 | 167 |
| *Tas2r124* | TGGTCTCCTTCCTCTTGCTC | GCTGCCTCATTACCCAAAGA | 59 | 95 |
| *Tas2r125* | AAAGGCCTTGCACATGGTAG | TCTTTGGACTTGCACAGCAG | 60 | 228 |
| *Tas2r126* | TGTTCTTTTGGGGAAACCAC | TGTGGGTATTGTGCTGCATT | 58 | 222 |
| *Tas2r129* | CATGTCTTTGGGCCTCTTGT | GCAGGAGAAAAGTGGACTGG | 59 | 200 |
| *Tas2r130* | GGACATGCCTCTGTCAAGGT | CTGCTTTCATGGCTTTCACA | 59 | 178 |
| *Tas2r131* | TGTGTCCCTAGCATCATTGC | CCGTGCTAACTGCATGAAAA | 58 | 181 |
| *Tas2r134* | TAGCTGCCTCCAGGTTCTGT | TCAGGGATCCCAGCAGTAAC | 61 | 249 |
| *Tas2r135* | TGCTCTCCTTCATCGTCCTT | CAGCAGCCCCTCTTTATCAC | 60 | 190 |
| *Tas2r136* | CTGGAGGAACCAATCCACCT | TTCTTTCAGCCAGAACCTTGC | 60 | 150 |
| *Tas2r137* | CCTCTGGCTCAAATGGAGAG | GTAGGCAACCAGGGAAACAA | 59 | 244 |
| *Tas2r138* | CGCTTCTCTCACACCTTTCC | CTGCTGGTTTGGGACTTCAT | 59 | 329 |
| *Tas2r139* | CAGCTCCAGCATGTTCTTCA | AGGAGGGAGTATTCCCGAAA | 59 | 154 |
| *Tas2r140* | TGGCACTTGTCATGCAATCT | AACTGGACTTCAGCCACCAT | 59 | 174 |
| *Tas2r143* | GGATGAGGAACCGGACACTA | TCCCAGAGGATGCCAATAAG | 59 | 151 |
| *Tas2r144* | GAGTTTTTCCAGGCTGTTGC | GCAGCAAACCAGAGGTTACAG | 60 | 150 |
| *Gnat3* | GAAGCTTCAGGAGGATGCTG | TCGGCTCCTAGGATTGACAT | 59 | 241 |
| *Plcβ2* | TGAAGCCATTGCAGAAAGTG | TTTTCCAGGGGATCTGTGAG | 58 | 153 |
| *Trpm5* | GGAACGACCTTTGGCTATGA | GGTGAAGGATTCGATCCAGA | 58 | 221 |
| *B2m* | ACCGGCCTGTATGCTATCCAGAA | AATGTGAGGCGGGTGGAACTGT | 60 | 120 |
