# Supplementary figures and images for: Alarm pheromone and kairomone detection via bitter taste receptors in the mouse Grueneberg ganglion
Source: BMC Biol. 2018 Jan 18;16:12. doi: 10.1186/s12915-017-0479-y (PMC5774136; doi:10.1186/s12915-017-0479-y)

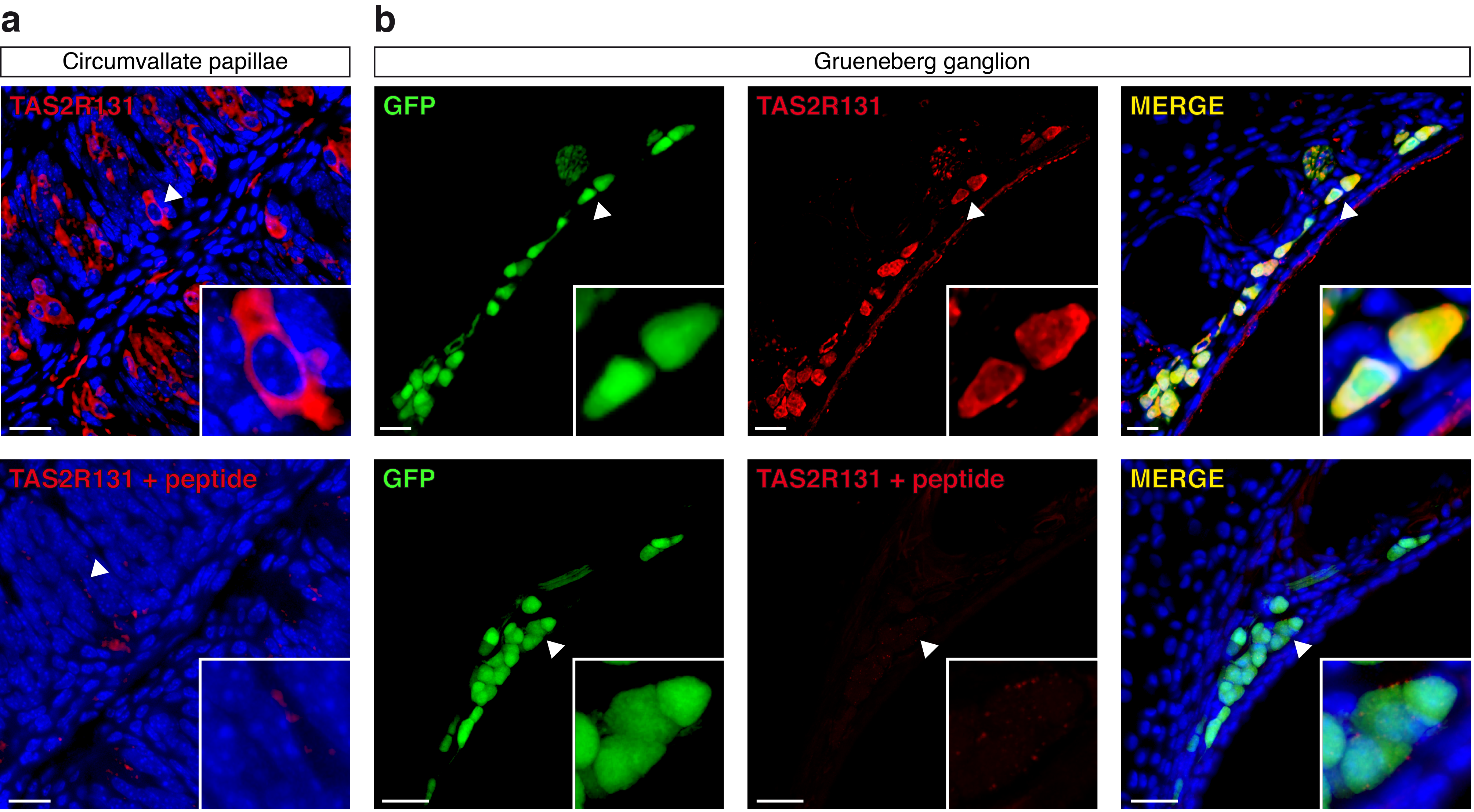

Supplement: Supplementary file 2 — Grueneberg ganglion neurons express TAS2R131. (a) Immunohistochemistries on mice tongue tissue section (circumvallate papillae) with antibodies against TAS2R131 with and without its competing peptide to verify the staining selectivity. (b) Immunohistochemistries on mice GG sections with antibodies against TAS2R131 with and without its competing peptide to verify the staining selectivity. GG cells expressing GFP are visible in green due to the intrinsic expression of the GFP. White arrowheads correspond to enlarged views displayed in insets; nuclei are counterstained in blue with DAPI; scale bars are 20 μm. (TIF 4156 kb) [file 12915_2017_479_MOESM2_ESM.tif]

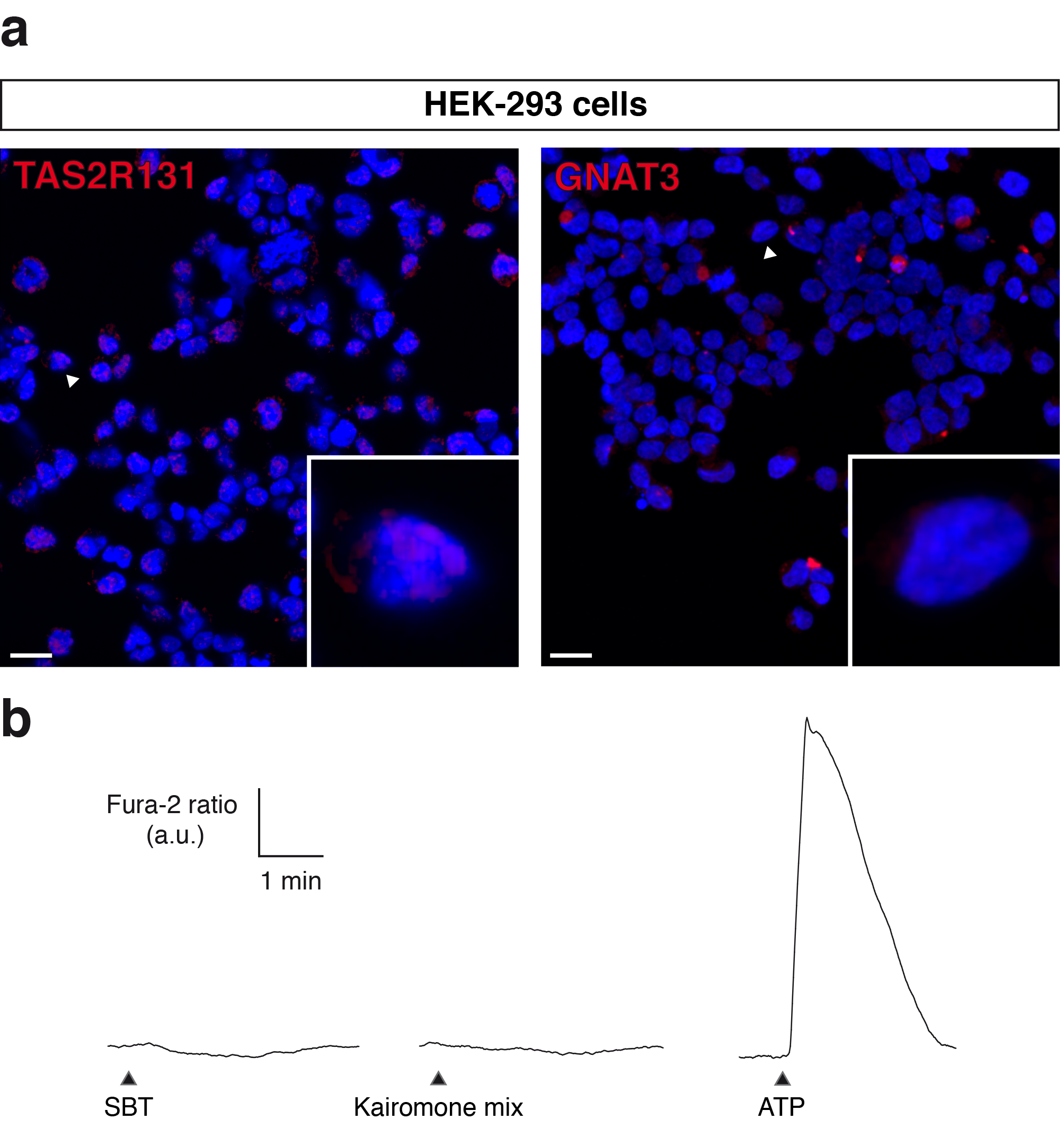

Supplement: Supplementary file 3 — HEK cells are not activated by Grueneberg ganglion ligands. (a) Immunocytochemistry experiments revealing the absence of expression of TAS2R131 and GNAT3 in HEK cells. (b) Representative intracellular calcium responses on HEK cells following perfusion of the alarm pheromone 2-sec-butylthiazoline (SBT) and of a mix of kairomones (TMT, 2-PT, 2,6-DMP and 2,4-Lu) at 100 μM. ATP was perfused as an internal control at 100 μM. In (a), white arrowheads correspond to close-up view of single HEK cell (insets). Scale bars are 30 μm. Nuclei are counterstained in blue with DAPI. In (b), fluorescence intensity Fura-2 ratio = F340/F380 is indicated by arbitrary units (a.u.); time is indicated by a horizontal bar. (TIF 1625 kb) [file 12915_2017_479_MOESM3_ESM.tif]
